# Design and implementation of a dental caries prevention trial in remote Canadian Aboriginal communities

**DOI:** 10.1186/1745-6215-11-54

**Published:** 2010-05-13

**Authors:** Rosamund Harrison, Jacques Veronneau, Brian Leroux

**Affiliations:** 1 Division of Pediatric Dentistry, Faculty of Dentistry, University of British Columbia, 2199 Wesbrook Mall, Vancouver, V6T 1Z3, Canada; 2 Public Health Department, Cree Board of Health and Social Service of James Bay, 200 Sam Awashish Street, Mistissini, Quebec, G0W 1C0, Canada; 3 Department of Biostatistics, Department of Dental Public Health Sciences, Box 359460, University of Washington, Seattle WA 98195 USA

## Abstract

**Background:**

The goal of this cluster randomized trial is to test the effectiveness of a counseling approach, Motivational Interviewing, to control dental caries in young Aboriginal children. Motivational Interviewing, a client-centred, directive counseling style, has not yet been evaluated as an approach for promotion of behaviour change in indigenous communities in remote settings.

**Methods/design:**

Aboriginal women were hired from the 9 communities to recruit expectant and new mothers to the trial, administer questionnaires and deliver the counseling to mothers in the test communities. The goal is for mothers to receive the intervention during pregnancy and at their child's immunization visits. Data on children's dental health status and family dental health practices will be collected when children are 30-months of age.

The communities were randomly allocated to test or control group by a random "draw" over community radio. Sample size and power were determined based on an anticipated 20% reduction in caries prevalence. Randomization checks were conducted between groups.

**Discussion:**

In the 5 test and 4 control communities, 272 of the original target sample size of 309 mothers have been recruited over a two-and-a-half year period. A power calculation using the actual attained sample size showed power to be 79% to detect a treatment effect. If an attrition fraction of 4% per year is maintained, power will remain at 80%. Power will still be > 90% to detect a 25% reduction in caries prevalence. The distribution of most baseline variables was similar for the two randomized groups of mothers. However, despite the random assignment of communities to treatment conditions, group differences exist for stage of pregnancy and prior tooth extractions in the family. Because of the group imbalances on certain variables, control of baseline variables will be done in the analyses of treatment effects.

This paper explains the challenges of conducting randomized trials in remote settings, the importance of thorough community collaboration, and also illustrates the likelihood that some baseline variables that may be clinically important will be unevenly split in group-randomized trials when the number of groups is small.

**Trial registration:**

This trial is registered as ISRCTN41467632.

## Background

The poor dental health of Aboriginal children in Canada is a major public health issue. Early childhood caries or ECC is the term used for dental caries in the young child. This condition can affect children before their first birthday [1]. ECC can be a painful condition, influencing a child's ability to eat properly, sleep through the night [2], grow and develop normally [3] and thus achieve full potential. Furthermore, caries in the primary (baby) teeth has a significant and positive association with caries and malalignment of the permanent teeth [4]. In some Canadian Aboriginal communities, the prevalence of ECC exceeds 90% [5]. Furthermore, the financial burden of treating ECC is enormous, and even more so for young Aboriginal children from remote communities who often must travel vast distances for comprehensive treatment [6].

Dental caries (tooth decay) occurs when "cavity-causing" bacteria, foods usable to the bacteria and susceptible teeth are in contact with each other long enough to allow bacterial by-products to demineralize the enamel of the teeth [4]. A universally effective, caries-prevention program with predictable long-term results for young Aboriginal children has yet to be found. Because ECC is a disease that is multi-factorial in origin, any preventive program must include a variety of strategies [7]. The microorganisms implicated in the initiation of dental caries are transmitted from mother to child [8-10]. The inoculation of bacteria received by the child appears to be related to maternal oral health status, diet and oral hygiene practices [10]. Once the baby's primary teeth begin to erupt, brushing the teeth regularly with toothpaste containing fluoride is essential. Another means of introducing fluoride is regular application of fluoride varnish[11]. Given that poor dietary behaviours also contribute to ECC, dietary modification is an important component of an infant oral health promotion strategy. A sleep-time bottle, constant daytime sipping from a bottle or sippy cup containing anything other than water and frequent snacking are practices linked to the development of extensive caries [12].

Most caries-prevention strategies require that a parent change an existing behaviour, for example bottle-feeding, or adopt a new behaviour, for example regular toothbrushing. However, providing knowledge alone to parents rarely leads to long-term changes in preventive behaviours [13]. Conversely, behavioural techniques that structure change and assist parents in the process of change may lead to long-term change in behaviours.

The purpose of this report is to provide the scientific rationale and methodological approach that were employed in a randomized controlled trial, involving Aboriginal (Cree) mothers and infants in eastern James Bay in Quebec, Canada. The goal of the trial was to test the effectiveness of an intensive one-on-one preventive counseling intervention, Motivational Interviewing (MI). Issues related to design of the trial, training and recruitment of staff, articulation with the existing organizational structure of health care delivery and implementation of the trial in these remote communities will be discussed. The aim of this report is to enlighten other communities and public health researchers who may be planning similar interventions.

## Methods

### Study-design

The research was approved by the Behavioural Ethics Review Board of the University of British Columbia and by the Cree Board of Health and Social Services of James Bay (CBHSSJB).

The trial was designed as a single-blind study with cluster randomization by community and two treatment groups. Randomization of individual mothers within each community was inappropriate for this study because of the close-knit nature of the communities and the risk of contamination. Therefore, participants were allocated to treatment conditions by community using cluster randomization. A cluster randomization design reduced the risk of cross-contamination but did not eliminate it completely. A characteristic of this project that reduced contamination was the fact that the nine "clusters" (communities) were distinct and well separated by considerable distance in many cases. Furthermore, each community had its own health clinic. In addition, few mothers of young children traveled much between communities because of the prohibitive cost and the difficulty of traveling with young children.

### Study-research questions

#### Primary question

Is there any difference in the dental health status of young Cree children whose mothers have participated in a client-centred, one-on-one, preventive counseling intervention, Motivational Interviewing (MI), compared with children whose mothers received oral health information in the form of an educational pamphlet?

This question will be answered by testing the hypothesis that the prevalence of caries in 30 month old children will be lower in the experimental communities than in the control communities.

#### Secondary questions

Are Cree mother's knowledge and beliefs about child dental health issues, their dental health practices and child feeding and comforting practices altered by participation in an intervention based on principles of Motivational Interviewing (MI)?

#### Additional questions

Will children whose mothers participate in the MI interventions have fewer negative health outcomes related to poor dental health, for example pain or problems eating, than control children? Does mothers' participation in these interventions decrease the probability of their children requiring dental treatment under general anesthesia or with sedation?

These questions will be answered by testing the null hypothesis that there will be no difference between the two groups.

### Study population

The Cree, an Aboriginal nation of North America, are the largest First Nations group in Canada with over 200,000 members. The Quebec Cree nation is called Eeyou Istchee - Cree for *Land of the People*, and lies to the east and southeast of James Bay. The Quebec Cree number some 14,000 people and live in nine distinct settlements.

In 1985, a survey of children in the eastern James Bay community of Chisasibi revealed that the mean number of decayed, extracted and filled primary tooth surfaces or the "defs" for 4-6 year old girls was 19.3 and for boys was 24.2 [14]. A more recent community-wide survey of 1079 Cree children between the ages of 12 months and 12 years [15] found that 30.4% of 12-24 month olds had dental caries. By comparison, only 4% of similarly aged non-Aboriginal children in Quebec had caries [15]. The amount of dental disease in these young Cree children is disturbing particularly given the fact that each Eeyou Istchee community has a dental clinic. It is unlikely that there will ever be enough dental clinics and dental practitioners in Eeyou Istchee to manage the amount of disease. Furthermore, Eeyou Istchee community water supplies have no added fluoride. Clearly, existing health services need to be supplemented with a population-based approach to promote improved child oral health.

### Inclusion/exclusion criteria

Over a period of about 2 1/2 years, all women in the 9 Cree communities who recently had given birth or were between about the 12^th ^and 34^th ^week of their pregnancy were asked to participate in the study. Any woman knowing of an impending, permanent move out of her community was excluded. Even children born with a medical concern or a congenital anomaly, for example cleft palate were included, if that was the family's wish. Information about the health of the child will be gathered at the outcomes assessment and will be managed in the statistical analysis.

### Study intervention: Motivational Interviewing (MI)

Patient-centred, personalized approaches that avoid direct persuasion have been shown to produce good results in promotion of behaviour change [16,17] The intervention in this trial followed the principles of Motivational Interviewing or MI [18], a client-centred but directive counseling style. With this approach, the motivation for change comes from the client, but the counselor helps create, by questioning and reflection, the expectation of change. Feedback and advice are offered within the context of acknowledgement of the client's right to choose. Many possible paths to a solution are provided. Client and counselor agree upon a menu of effective behaviours. This strategy fits well with the philosophy of the Cree who are more comfortable if someone suggests ways to think of taking a different approach rather than tells them directly how to act. It also fits well with a recommendation from a gestational diabetes program in Eeyou Istchee that, for an intervention to be effective, women should be given the opportunity "to reflect on their roles as women and mothers, caregivers and providers" [19].

MI has been successful in the management of addictive behaviours such as smoking and non-addictive behaviours associated with conditions like diabetes [16,17]. Furthermore, brief MI interventions have produced good results [20]. MI has been previously successfully applied in a trial to reduce ECC in 6-18 month old IndoCanadian children in western Canada [21]. At the end of the 2-year trial period, the MI children had a 46% lower rate of tooth surfaces affected by caries than did control children.

In this project called "I wish my child would have beautiful teeth" or, in the Cree language, *Kimaa Miywaapitet Nitawaashiim *experimental group mothers had an MI session during pregnancy and, ideally, participated in several more MI sessions until their child was two years of age. Mothers received appropriate resources at each MI visit to enable them to implement selected behaviours (infant toothbrushes, toothpaste, sippy cups.) Fluoride varnish was offered after the age of one-year.

The control group mothers received a culturally-appropriate educational pamphlet describing healthy dental care practices for young children. Pamphlets were mailed to mothers when their child was 6 months of age and again at 18 months of age. The pamphlet titled "Protect Baby Teeth: Circle of Smiles" had been previously produced in 2000 by the Nursing Caries Committee of the St. Theresa Point First Nation of Manitoba, Canada and is available from them on request [22]. Fluoride varnish was available to control children at local dental clinics.

### Randomization

The advantages and disadvantages of testing the intervention as a randomized controlled trial were discussed and debated at length during a 2-year community consultation process. Those who participated in the discussions during the consultation's phase were reassured that the randomization process would be open, impartial and unbiased. Therefore, the randomization was done publicly during a daytime broadcast over community radio. The territory's community radio station with its extensive listening audience was an accepted and trusted way of conveying local news and information in Eeyou Istchee. A community radio broadcast of the randomization process was well-suited to the remoteness of Eeyou Istchee and the distances between communities.

There were two "rounds" of a constrained randomization process: one round for the 2 larger communities and a second round for the 7 smaller communities. Two baskets had been prepared for the "on-air" randomization: one basket for the large communities and another basket for the smaller communities. Each basket contained envelopes marked "test" or control"; the larger community's basket contained 2 envelopes (1 test; 1 control) and the smaller community's basket contained 7 envelopes (4 test; 3 control). Communities were randomized in each round by alphabetically ordering the communities' names. For example, for each round the first name on the alphabetical list of communities was announced followed by the drawing of an envelope from the basket; the next name was announced followed by another draw until all envelopes were allocated. The draw was done "live" on afternoon radio by a radio station employee who was not associated with the research. Of the 9 communities, 5 were allocated to the test condition and 4 to the control condition. The decision was made to allocate one more test than control community to allow a more robust exploration of intervention effects e.g. analyses according to number of MI sessions attended. Communities were not aware of their allocation until their name was drawn. No concerns about the randomization process have been expressed by any community.

### Study personnel

Cree dental assistants who worked part-time in community dental clinics were ideal personnel to work on this project. The study protocol suggested that some of these dental assistants be recruited and employed in a new capacity as Dental Health Representatives, or DHRs, to work on the project in test communities and also to recruit mothers from neighboring control communities. Training and calibration in recruitment procedures and in the MI technique were to be provided to the DHRs in workshops facilitated by an expert in MI. DHRs would learn how to frame questions, help structure change, apply fluoride varnish and demonstrate toothbrushing. Instruction and practice in administering the survey instruments would also take place.

### Instruments

#### Questionnaires on oral health practices

Women enrolled in the study completed instruments that included items on demographics, their personal oral hygiene practices and dental knowledge. An instrument called the Readiness Assessment of Future Parents concerning Infant Dental Decay or RAFPIDD was also completed. This 47-item instrument was a modification of a validated instrument developed by Weinstein and Riedy to assess a mother's stage of change with regard to her child's dental health [23]. The instrument was enhanced with items specific to Cree mothers and pre-tested for internal consistency with pregnant women in Eeyou Istchee.

#### Child dental health and dental health behaviours at end-point

Clinical data will be collected by a dental examination of each child at 30 ± 3 months of age. Calibrated examiners from outside of Eeyou Istchee who have no other association to the trial will do the assessments. The clinical detection of caries will involve visual/tactile examinations with explorers to remove plaque, front surface mirrors, cotton rolls and a dental head light. Criteria for caries detection will be those described by Pitts [24]. Ten percent of the children will be re-examined on a random basis for assessment of reliability.

Information about dental health knowledge, home-care behaviours and caries-related health impacts will be collected by survey instruments administered at the assessment visit. The survey questions have been validated in a previous survey of 301 children undertaken in community health clinics in Quebec [15].

#### Economic evaluation

The objectives of the economic evaluation will be to estimate the net incremental cost of the intervention strategy and to estimate an incremental cost-effectiveness ratio. Estimation of the net incremental cost of the program will involve a comparison of costs of dental resources utilized by each arm of the study. The cost analysis will involve a study of the time and materials required to carry out each of the intervention visits. Salary costs for the DHRs will be assessed based on their compensation rate and disposable materials will be valued at acquisition costs. Value of the mother's time to take her child to the clinic for well-baby care and for MI will be determined by age and gender-matched wages and subjected to sensitivity analyses. If the intervention is effective, children in the intervention arm should have lower rates of use of dental-related health services during the period of the trial. Information concerning types, amount and cost of care received as well as the time lost by caregivers in pursuing this care will be collected. The net cost of the program, then, will include the intervention costs minus any savings in dental services. The incremental cost-effectiveness ratio at follow-up will be based on these net incremental costs and the difference in the primary outcome measure, number of decayed, extracted or filled primary tooth surfaces.

#### Sample size

The target sample size of 309 mothers was determined based on the need to have high power to detect a 20% reduction in caries prevalence. This magnitude of reduction was similar to that observed in a community-based oral health promotion program implemented in Native American villages that demonstrated a 25% decrease in "baby bottle tooth decay" in participating communities after 3 years [25].

The power was calculated using the method of adjustment for intra-class correlation by the variance inflation factor [26] applied to the standard formula for calculating power for comparison of proportions from independent samples. The intra-class correlation coefficient was estimated to be 0.0090 by applying the analysis of variance method to the preliminary data on caries prevalence by village, which produced a variance inflation factor of 1.35. The control-group caries prevalence was estimated to be 0.86 using a weighted average of these individual prevalences weighted by sample size per village. Based on the above projections, a total sample size of 265 mother and child pairs would yield power of 82% to detect a 20% reduction in caries from 0.86 to 0.69.

A reported infant mortality rate of 15 deaths per 1000 births was considered [27]. Overall loss to follow-up, including mortality, was estimated to be 5% per year for the 3 years that mother and baby were in the study. Thus, the sample size was determined to be 309 mother and child pairs. Based on a 75% participation rate, 412 births were required to achieve a sample size of 309. Other researchers working in Eeyou Istchee with new mothers have reported an 80% participation rate which was similar to our expectations. The anticipated number of children enrolled in each community was estimated using the birth data from 2001 and a 75% participation rate in each village, for a total sample size of 258 accrued per year. Thus, it was estimated that 14 months would be required to enroll 309 mothers.

### Statistical analysis

#### Primary statistical analysis

The primary statistical analysis will be a comparison of caries prevalence in intervention and control groups, using a permutation test[28] with test statistic equal to the difference between caries prevalences in the two groups. A significance level will be determined using the exact permutation distribution of the test statistic, which will be computed by enumerating all possible random assignments of villages to intervention or control conditions according to the randomization scheme. The use of the permutation test accounts for intra-class correlation between outcomes on children in the same village and also addresses concerns over the small-sample performance of statistical methods such as Generalized Estimating Equations [29], which rely on asymptotic theory. An exact confidence interval for the treatment effect (difference in caries prevalences) will be computed using the usual procedure for inverting the permutation test. Peterson et al provide an illustration of the application of this procedure to a group-randomized trial [30]. Additional analyses will be conducted using the same procedures for boys and girls separately.

The main analysis will be done at the end of the study when data collection at 30+/-3 months is complete. No interim analyses will be performed because it is considered extremely unlikely for there to be evidence of a treatment effect prior to collection of the final outcome data on the entire sample. However, a preliminary frequency analysis of the baseline data was done to help the investigators develop an understanding of the characteristics of the sample.

### Subgroup analyses

Within the experimental communities, outcomes will be compared looking at factors such as total number of MI sessions attended, number of fluoride varnish applications, individual DHR and individual community. Within the entire study sample outcomes will be compared looking at factors such as number of fluoride varnish applications and reported frequency of tooth brushing.

### Progress of recruitment and power

Two hundred and seventy-two mothers (131 test; 141 control) have been recruited over 2 1/2 years. Because of the cluster-randomized design of the trial, the power of the trial can be determined not only by the total sample size but also by the number of communities and by having sufficient numbers of subjects recruited in each of the communities. Whereas the original projected sample size of 309 women would have yielded 82% power to detect a 20% reduction in caries prevalence from 0.86 to 0.69, power calculations using the actual sample sizes show that the power will be 79% to detect this treatment effect. These calculations assume a loss to follow-up of 5% per year. If we are able to maintain an attrition fraction of 4% per year, the power will be 80%. Power will be very high (> 90%) to detect a 25% reduction in caries prevalence.

### Baseline characteristics

For the entire group of women recruited, the mean (SD) age was 25.6 (6.1) years; stage of pregnancy was 20.2 (10.7) weeks. The majority or 65.1% of the women had other children and 42.0% of these mothers had a child who had previously had a tooth extracted. The women's mean (SD) score out of 5 for the knowledge questions was 3.0 (1.3). As far as oral health behaviours, 92.3% of mothers brushed their teeth with toothpaste at least once a day and 72.7% of mothers had been to a dentist within the last two years.

### Treatment group comparisons at baseline

Randomization checks were conducted on possible demographic and behavioral differences between groups (Table 1). The distributions of most variables was very similar for the two randomized groups of mothers (specifically, for age, knowledge score, other children, toothbrushing, and seeing the dentist in the prior two years). However, despite the random assignment of communities to treatment conditions, group differences existed for stage of pregnancy and prior tooth extractions for other children in the family. Mothers in the test or MI communities had later stages of pregnancy than mothers in control communities (22.4 vs. 17.7 weeks), and mothers with other children in the MI communities were less likely to have had another child with a tooth extraction (34 vs. 49%). Testing of differences between randomized groups on baseline variables is not particularly informative, because any differences found must by definition be type I errors. However, such tests were performed here for illustrative purposes. Note that the difference in pregnancy stage did reach statistical significance (Z-statistic larger than 2 corresponds to a p-value < 0.05), whereas the difference in tooth extraction prevalence did not. These results illustrate that a difference that could be deemed clinically important may result even within the range of normal sampling variability in the cluster-randomized setting. In contrast, such a difference might be less likely to occur in an individual-randomized design with the same number of participants. Statistical significance aside, what matters is that the group differences (particularly for tooth extraction) may be large enough to be associated with clinically meaningful differences in health outcomes at follow-up in the enrolled children. Therefore, secondary analyses of outcomes will be done with regression adjustment to control differences in stage of pregnancy and presence of other children with tooth extractions as covariates. An unadjusted analysis will be performed as the primary analysis; the results of the adjusted and unadjusted analyses will be compared to determine the impact of the baseline group differences on the results.

**Table 1:** Randomization effectiveness: baseline characteristics of study sample mothers by treatment group.

Variable	N	Test	N	Control	Z^1^
Age (years)^2^	130	25.5 (6.4), 15 - 44	137	25.6 (5.8), 15 - 39	-0.11
Stage of pregnancy (weeks)^2^	102	22.4 (8.7), 6 - 38	90	17.7 (12.1), 0 - 36	2.25
Knowledge score^2^	127	3.1 (1.2), 0 - 5	140	2.9 (1.4), 0 - 5	0.57
Has other children^2^	129	83 (64.3%)	140	92 (65.7%)	0.32
Other child had prior tooth extraction^3,4^	82	28 (34.1%)	92	45 (48.9%)	1.09
Brush with fluoride toothpaste^3^	130	120 (92.3%)	141	130 (92.2%)	0.04
Saw dentist < 2 years ago^3^	130	93 (71.5%)	141	104 (73.8%)	-0.42

^1^Z = observed Z-statistic for comparison of MI and Control groups using Generalized Estimating Equations to account for clustering of subjects within communities

^2^Results shown are mean (SD), minimum - maximum

^3^Results shown are number positive for the variable (% positive).

^4 ^Only responses of mothers with other children are included

## Discussion

This project with Aboriginal mothers aimed to evaluate the effectiveness of a specific behavioural intervention, Motivational Interviewing, to decrease the prevalence of early childhood caries in their children. The location of the trial in remote communities in northern Canada added to the project's challenges.

### Community consultation

The process of community consultation was one of several complex issues that arose during the planning of the trial. Prior to undertaking the trial, extensive discussions throughout the community were undertaken. While disturbances in the balance between bacteria, substrate and host are the local factors associated with ECC, health determinant like economics, social norms and housing conditions also have a substantial impact on the development of the disease [31]. Therefore, any intervention had to be planned with early and continuous community collaboration and consultation and needed to be sensitive to existing beliefs and traditions about parenting practices, child comforting and infant health. Oral health intervention strategies must be sensitive to the intended target population because of the important role that culture plays in shaping health-related attitudes and behaviours in Aboriginal communities [32]. The project investigators, one of whom was the public health dentist for the territory, undertook two years of consultation with community leaders, health care workers and families from Eeyou Istchee to discuss appropriate strategies prior to beginning the trial. In addition, the Research Committee of the CBHSSJB collaborated on this project from its conception.

When the project began, relatively few community-based initiatives to improve the oral health of young children had been undertaken [25,33] and scant few had been conducted as randomized controlled trials [34,35]. The concept, advantages and disadvantages of a randomized controlled trial were discussed at length to ensure that communities understood that, during the trial, "test" communities were to have a more intensive program than "control" communities. The consensus from the consultation was that, since no infant oral health promotion program was currently in place, it was acceptable to participate in a trial to definitively determine the most effective approach.

### Existing organizational structure

Another matter that arose during the planning of the trial was how the project was to articulate with the structure and organization of health care services within Eeyou Istchee. Surveillance, promotion, prevention, protection, regulation, research and training relating to the health of the Cree population in Eeyou Istchee are managed by the Public Health Department of the CBHSSJB. The Department's long-term goal related to this specific project was that, if successful, the MI approach would become a component of the Department's health promotion activities. To that end, their inclination was for the day-to-day work of the project to be undertaken by existing Health Department staff rather than new staff, the so-called "Dental Health Representatives" (DHRs), who were to be specific to the research project. After considerable deliberation, it was decided that existing department staff, women currently employed as Community Health Representatives (CHRs) would do the recruiting of mothers and be the project interveners. The Project Manager who was hired was a dental hygienist from the Cree nation who was born, raised and had worked for several years in Eeyou Istchee.

### Training of project staff and the MI intervention

One of the first steps in launching the project was staff engagement ("buy-in") and training. Because of the decision to engage existing staff (CHRs) as recruiters and interveners in the project, job-postings and hiring did not slow down the start of the project. However, 3 of the 9 communities did not have a CHR on staff at the project's start; therefore, local women (as per the original proposal) were hired to begin recruitment. From the beginning of the project and throughout, the Project Manager visited the communities and met with the project's staff to problem-solve challenges and review procedures. She also maintained regular telephone contact with the staff in each community who were working on the project.

As detailed in the proposal for the trial, a two-day training workshop for CHRs in the 5 intervention or "test" communities was held within the first year of the project. The project's MI consultant provided a template for the MI script and menu that would be used in the counseling sessions with the mothers. By engaging the CHRs in discussion and critique, both the script and the menus were extensively modified to suit the language and style of the Cree. Following the workshop, the menus were finalized, printed on flipcharts and sent with explanatory notes to each CHR in each of the test communities. The MI consultant followed up some months later with an "MI-coaching conference call" to problem-solve MI with the CHRs. A second follow-up workshop was held the next year. Following this workshop, the PM visited each of the communities individually to problem-solve recruiting and MI challenges.

The MI scripts were based on the work of Weinstein[36] and on scripts developed for a previous trial [21,37,38] One script was created for pregnant and new mothers (Additional file 1) and another slightly modified script was developed for mothers after their infant's first tooth had erupted until their child was about 2-years of age. Mothers of newborn, pre-dentate infants were recruited to the trial in addition to pregnant women; this was a variation from the original proposal but was done to ensure that every newborn infant and mother had the opportunity to participate. An important aspect of improving infant oral health is enhancing the oral health of the mother to prevent transmission of cavity-causing bacteria from mother to infant. With this in mind, pregnant and new mothers were given "privilege cards" that allowed them expedited dental services at their community's dental clinic.

For a variety of reasons, CHRs in many of the communities were challenged by the process of subject recruitment and delivering the intervention. The negative side of engaging these existing personnel in a research project was their sense of being overworked and not sufficiently rewarded for this addition to their daily workload. This project was yet another burden for the already busy and in-demand CHRs. Furthermore, the CHRs may have found that expectant women and new mothers were too busy to spend an additional amount of time completing project documents or were simply not interested in participating in a research project. Even though the project investigators had tried to be diligent in following recommended principles of involving Aboriginal communities in research, recruitment of participants was a struggle. Thus, in 2 of the 5 test communities and in 2 of the 4 control communities, recruitment and, for the test communities, delivery of the intervention was eventually completed by the Project Manager. One of the project-specific DHRs achieved success in one test-community and eventually took over the work of a retiring CHR in another test-community. A CHR, aided by the Project Manager, completed and continued the project's work in the 5^th ^test community. Local women who were not CHRs completed recruitment in the remaining 2 control-communities. Recruitment was closed after 2 1/2 years when the number of mothers recruited, though short of the original goal, was sufficient to maintain the power of the trial.

Administrators may request that in order for research trials to be implemented within their organization, existing personnel should work on the project. However, these individuals may simply not have the time, the interest or the desire to participate, despite the provision of extensive in-service training and ongoing support. Therefore, it can be concluded that in these instances, the trial's human and financial resources need to be efficiently and quickly redistributed to ensure success of the project. Despite the initial and ongoing challenges involved with implementing this clinical trial, the results will enhance our understanding of the details of implementation of randomized trials in remote communities. In addition, the role of a client-centred, directive counseling style like Motivational Interviewing in enhancing positive oral health behaviours in Aboriginal families will be better understood.

## Competing interests

The authors declare that they have no competing interests.

## Authors' contributions

RH and JV obtained funding for the study. All authors contributed to the design of the study. RH and JV developed the instruments, adapted an MI counseling protocol to the study and supervised the collection of baseline data. JV oversaw all phases of the community consultation. BL performed the sample size calculations and the analysis of the baseline data. RH wrote the manuscript. All authors have read and approved the final manuscript

## Supplementary Material

Additional file 1 Motivational Interviewing Script. Click here for file
