# Supplementary material for: Design and implementation of a dental caries prevention trial in remote Canadian Aboriginal communities
Source: Trials. 2010 May 13;11:54. doi: 10.1186/1745-6215-11-54 (PMC2887856; doi:10.1186/1745-6215-11-54)
Supplement: Additional file 1 — Motivational Interviewing Script. [file 1745-6215-11-54-S1.DOC]

**“I wish my child would have beautiful teeth”**

***Kimaa Miywaapitet Nitawaashiim***

**Motivational Interviewing Script**

**(For pregnant woman or new moms before first baby tooth comes in)**

You might need to say to a mom-to-be or new mom at the first session:

- *“It may seem early, but I would like to talk about your new baby’s teeth”*
- *“What we say to each other will be confidential”*
- *“I will be looking at my guidebook and taking notes as we talk” or, if mom will be more comfortable, make notes after she leaves*

### Visit #1: During pregnancy

### Visit #2: At 2- or 4-months immunization appointment

**Background for pregnancy and new moms before first baby tooth comes in**

*Why is it so important for mom-to-be or new mom to have a healthy mouth?*

For a cavity to start, at least 3 factors are required

1. Sugary foods and/or drinks

2. Cavity-causing bacteria (germs)

3. Susceptible teeth

Babies are not born with cavity-causing bacteria (germs*) in their mouth

**These germs are usually passed from a mom to her baby*

*How does a mom spread these germs to her baby?*

If mom has cavities that have not been fixed, or does not keep her teeth as clean as she should, she will have cavity-causing germs on her teeth and in her saliva (spit)

Therefore, whenever a mom

-kisses her baby

-licks a soother to make it clean

-blows on her baby’s food

-or, any of those good things that we expect moms to do, she is at risk of passing on these germs to here baby.

*How does a mom stop spreading these germs to her baby?*

A mom should have all her cavities fixed and brush her teeth regularly. Cutting down on sugary foods and drinks and chewing xylitol-containing gum will help stop new cavities from forming in her mouth.

That is why the themes of the counseling that you will do ***before*** a baby gets any teeth are:

**1. Improving Mom’s oral health**

**2. Stopping the spread of cavity-causing bacteria (germs) from mom to child**

1. ASK QUESTIONS: **WHY ASK QUESTIONS?**

1. To show your concern or empathy for mom and her new baby
   - Counseling, like motivational interviewing, works best when you have developed a relationship with a mom
     - when you know her, even a little bit
2. To get mom to talk, so you are not doing all of the talking
   - You should do more listening than talking

- Only ask the type of questions that you are comfortable with

An indirect question might be best! For example

- *”Some parents have had problems with their own teeth, so they worry about their children’s teeth…what about you?*
- Or you could ask other questions, just to get mom talking
- “*Do you have other children*?” (write down children’s names and ages)
  - Comment about other children, for example, *“It must be hard for you to have time for yourself as well as look after……”*
  - Encourage the mother to tell you about the pregnancy. For example,
    - *“Are you sleeping and eating okay?”*
    - *“Do you work outside of the home?”*
  - Respond to the mother by nodding and paraphrasing. Encourage her *“Tell me more.”* Write down important points in her folder.
- At our workshop, we decided not to dwell on the past, but look to a positive future.
- You also felt that moms may not want to talk (with you) about their own, or their other children’s teeth especially if there have been “problems”
- So, we will move on to asking what a mom wants for her new baby’s teeth
- Ask mom what she wants for her child’s teeth
  - *“If we could change the future, leaving the past behind, tell me what you would want for the dental health/teeth of your child?”*
  - *“Tell me more.”* or *“Anything else?*”______________________.
  - Repeat back to mom what she has just told you. This lets her know that you have been listening!
    - *“Let me be sure that I understand. You would like your child to………”*
  - Write down what she wants: ____________________________________________________

2. BE POSITIVE ABOUT HOW GOOD A PARENT SHE IS TO WANT THESE THINGS FOR HER NEW BABY.

- *“You are/will be a really good mom”*
- *“You really love that baby.”*
- *“You are really good at…*.”

3. SAYING IT AGAIN MAKES IT SO!

- that is, say things to motivate mom
- “*I think I heard you say you want your baby to have great teeth. Did I get it right?”*
- *“Your child will thank you (for doing this).”*

4. REFLECT, LISTEN AND SUMMARIZE!

- - summarize dental health wish here
- *“Please tell me again what you want for your child’s dental health, so I can be sure I get it right”*

5. TIME TO MOVE ON TO THE MENU

Providing information to a parent who is ready to do something:

- - *“In order to (repeat again mom’s dental wish), I want to share with you some things we have recently learned.”*
  - *“Would you like to hear about them?”*

Ask her permission!

- - *“We have spoken to many moms about what we can do to help our children have healthy teeth. They suggested that we talk to other moms during pregnancy or before the baby gets her/his first tooth. That is why we are with you now.”*
- *“Some of the steps that were suggested for moms are on a list (menu) that I would like to show you.”*
  - Explain to mom that if her teeth are healthy, her baby will have a better chance of healthy teeth**. (Background in box on page 1.)**
  - Emphasize choice: no need to choose everything*!*
- Show mom the menu*.*

**Menu for pregnant woman or new mom before first baby tooth comes in**

1. Brush my own teeth with toothpaste at least once a day.
2. If it has been more than 6 months since I went to the dental clinic, I will make an appointment for a check-up.
3. I will have healthy meals and snacks in my diet
4. I will drink less pop or drink pop only with meals.
5. I will only chew sugar-free gum.
6. I will always hold my baby during feeding, then lay him/her down to sleep and, if he/she wakens, I will give water, not formula or juice
7. I will sometimes give my new baby a soother if he/she is fussy, not a bottle.
8. Other______________________________________

**If time permits, do the following or go on to #6 and do this next visit:**

a. Review the menu items

*“Let’s look at the items on the menu, talk about each of them briefly, and you decide which ones are for you.”*

The following is an example:

*“Is it possible for you to brush your teeth with toothpaste at least once a day?*

*“What problems would you face if you tried to do this?” E.g. lack of time*

b. Review the rest of the list:

- *“What problems would you face if you tried to do any of the others that you have chosen?”* Examples of problems below
  - fear of the dentist,
  - lack of time
  - hard to get an appointment at the clinic
  - good food is too expensive*!*

c. Say, “*Maybe you have some ideas of your own on what to do?*

Be positive about any idea. “*That is a great idea!*”

d. Identify additional benefits

- - *“Let’s talk about the additional benefits of each item you have chosen.”*
  - *“Any other good things that would happen when you for example,*
    - Change snacking habits— I will be healthier (less weight problems, diabetes, heart problems);
    - Cut down on pop (I will save money)
  - “*Is this what YOU want to do?”* _____

6. INCREASE MOM’S BELIEF IN HERSELF THAT SHE CAN DO THIS

- Say supportive words: ***“I can tell that you are/will be a great parent/mom…”***
- Highlight her competence and abilities: ***“I can tell that you are/will be a great parent/mom…”***
- Encourage her to tell you again what she wants for her child: ***“Tell me again what you want for your child’s teeth/smile?***
- Remind her of outcome if she did nothing: ***“If someone chose not to do anything to help a child have healthy teeth, what would happen?”***

7. IF MOM IS “RESISTANT”: TIME TO CHANGE YOUR APPROACH!

- If a mom argues, interrupts, or shows reluctance. Do not argue with her
  - Try something different, for example,
    - Emphasize choice.
    - Agree with mother: *“It’s your choice. I’m not here to make your decision”*
  - Shift into reverse—that is, go back to the beginning
    - Work on rapport and trust: *“I really believe in you, your child will have great teeth because of you”*
    - Ask questions, listen
  - If mother is not ready to make commitment do not press her; say,
    - “*If you are not quite ready yet to take the steps we have gone over, I do not want you to go ahead and make a commitment. This is too important to decide now.*
    - *“Go home and think it over.”*
    - *“I will see you when you bring in your new baby for his/her 2-month immunization, and we can talk about it further.”*
    - *“I hope everything goes well for you.”*

8. WHAT TO SAY WHEN YOU HEAR:

“Baby teeth are not important.”

“All kids get cavities; it is normal….”

“My family doesn't have strong teeth; our teeth decay/go bad easily.…..”

- Listen to what mom is saying.
   *“I understand what you are saying; we used to believe that too…”*
- Say, as we discussed “new knowledge exists”:
   *“Recently research has given us new information/approaches…”*
- Remind mom about what you said about mom spreading germs to baby, etc.

9. ENDGAME

- Give a copy of the menu to mom

*“I am going to give you a copy of your menu”*

*“The items that you chose are checked off”*

*“You know where to contact me if you have more questions”*

- Anticipate problems

*“Not everything goes the way we plan.*

*“There are always problems.”*

- Encourage contact with CHR

*“Feel free to call me if you have any problems with your men”*

*“I appreciate your situation and your willingness to try.”*
